# Medicine and management: looking inside the box of changing hospital governance

**DOI:** 10.1186/s12913-016-1393-7

**Published:** 2016-05-24

**Authors:** Ellen Kuhlmann, Ylva Rangnitt, Mia von Knorring

**Affiliations:** Medical Management Centre, LIME, Karolinska Institutet, Stockholm, Sweden; Institute of Economics, Labour and Culture, Goethe-University Frankfurt, Frankfurt, Germany; Division of Insurance Medicine, Department of Clinical Neuroscience, Karolinska Institutet, Stockholm, Sweden

**Keywords:** Medical management, Hybrid hospital governance, Doctors in management, Actor-centred healthcare governance, Multi-level governance, Needs of managed and managing doctors, Sweden

## Abstract

**Background:**

Health policy has strengthened the demand for coordination between clinicians and managers and introduced new medical manager roles in hospitals to better connect medicine and management. These developments have created a scholarly debate of concepts and an increasing ‘hybridization’ of tasks and roles, yet the organizational effects are not well researched. This research introduces a multi-level governance approach and aims to explore the organizational needs of doctors using Sweden as a case study.

**Methods:**

We apply an assessment framework focusing on macro-meso levels and managerial-professional modes of hospital governance (using document analysis, secondary sources, and expert information) and expand the analysis towards the micro-level. Qualitative explorative empirical material gathered in two different studies in Swedish hospitals serves to pilot research into actor-centred perceptions of clinical management from the viewpoint of the ‘managed’ and the ‘managing’ doctors in an organization.

**Results:**

Sweden has developed a model of integrated hospital governance with complex structural coordination between medicine and management on the level of the organization. In terms of formal requirements, the professional background is less relevant for many management positions but in everyday work, medical managers are perceived primarily as colleagues and not as experts advising on managerial problems. The managers themselves seem to rely more on personal strength and medical knowledge than on management tools. Bringing doctors into management may hybridize formal roles and concepts, but it does not necessarily change the perceptions of doctors and improve managerial–professional coordination at the micro-level of the organization.

**Conclusion:**

This study brings gaps in hospital governance into view that may create organizational weaknesses and unmet management needs, thereby constraining more coordinated and integrated medical management.

## Background

Health policy has introduced new forms of hospital governance, which attempt to combine different sets of governing to improve organizational efficiency and accountability of professionals [1–3]. The reforms have created a qualitatively new demand for collaboration and coordination between clinicians and managers and introduced new medical manager roles in hospitals. Following an initial debate of either positive or negative effects, often based on dichotomous concepts of management and medical professionalism, research is getting increasingly more complex (for an overview see, [4–6]). One important strand of scholarly work has highlighted organizational and managerial connections and an emergent ‘hybridization’ of medicine and management [7–16], while other studies have primarily explored how doctors respond to the new managerial tasks [17–21]. Few authors have applied a cross-country comparative perspective [2–4, 22–26] or explored connections between professions in management and service outcomes [27–29]. However, there is still little research into the dynamics and effects of the new connections between medicine and management in every-day work of hospital doctors and how these changes impact and create novel demand for management to support doctors in their new roles.

This study seeks to contribute to the debate by placing changes in clinical management in a broader framework of hospital governance to explore the needs of doctors in the organization using Sweden as a case study. We apply an assessment framework, which was developed in a European comparative study with a focus on coordination between medicine and management on macro- and meso-levels [26] and expand the focus towards the micro-level of the organization. The micro-level approach includes the perspectives of the ‘managed doctors’ (rank-and-file doctors working in patient care), and the ‘managing’ doctors (the doctors with management responsibilities). The aim is two-fold: to contribute to the development of theory-led approaches and assessment tools in clinical management research [4, 5, 30, 31] and to apply the Hospital Control Assessment Framework tool [26] to the Swedish case in order to explore gaps and organizational weaknesses that may constrain new forms of more integrated (or hybrid) clinical management.

The Swedish healthcare system [32] is an interesting country case to look inside the box of changing clinical management, because of its strong institutional integration of the medical profession in the healthcare system and the structural integration of doctors in hospital management through new manager roles. Furthermore important, in Sweden the substantive organizational restructuring and the ‘managerial turn’ in hospital governance already started in the early 1980s, even slightly earlier than in England (for more general information, see [32–35]). The Swedish case may therefore be helpful to explore effects of new forms of clinical management, which are emerging more slowly over time and may not show up immediately after new models and structural changes have been implemented.

Especially interesting is the question, whether and how integrated hospital governance with complex modes of coordination between medicine and management on the meso-level of the organization impacts further down on the micro-level of doctors’ perception of management. This question will guide our research.

Clinical management and multi-level governance are umbrella terms, which may have different meanings. In this study the terms ‘clinical’ and ‘medical’ management are used interchangeable, while the focus is on hospital doctors in their roles as managers and as rank-and file doctors who receive managerial advice. Accordingly, ‘medical’ and ‘clinical managers’ describe doctors with management responsibilities and jobs. The concept of governance [36], as applied here, includes different hierarchical levels and different modes of governing, such as professional and managerial tools for instance. This multi-level approach to governance [37] allows us to explore connections and gaps in governance arrangements [38].

Following the definition used by Saltman and colleagues in their comparative study of public hospitals in Europe, three (hierarchical) levels of governance interact ‘with each other in complex patterns that then define the actual “governance structure” for hospitals’ ([2], p4). According to this definition, the macro-level of governance is part of the traditional dimensions of national/regional/supra-national policy-making (e.g., finance, structure and organization of hospitals). Meso-level governance is mainly focused on the decision-making at institutional levels of the hospital, while the micro-level refers to the everyday operational management with its traditional areas of management ([2], p5).

The definition is useful but needs further investigation for our purpose in order to explore *how* managerial and professional modes of governing are coordinated at the organizational level. In the next section we introduce an assessment tool (Hospital Control Assessment Framework; http://www.cies.iscte-iul.pt/np4/?newsId=632&fileName=COST_WG2_H_CAF_online.pdf, [26]) specifically developed to explore the coordination between medicine and management.

## Methods

The research is qualitative and explorative in nature and applies a case study design informed by a multi-level governance approach. The hospital assessment framework is applied to Sweden (with a focus on the meso-level of the organization) and combined with in-depth interview material from two different studies, which serve to pilot micro-level organizational effects of changing hospital governance.

The Hospital Control Assessment Framework (H-CAF) has been developed in European comparative research (not including Sweden) and described at length elsewhere [26]. It comprises the following five major categories of organizational and professional governance and their connections with a focus on the meso-level of governance.

1. ‘Key characteristics of the healthcare state and institutional contexts of hospital governance (macro-level);
2. governance structures of the hospital (meso-level/organisation);
3. financial/efficiency controls and managerial tools (organisational levels, accountability and actors);
4. quality and safety controls and organisational-managerial tools (organisational levels; accountability and actors);
5. professional/medical self-governing controls and tools (organisational levels; accountability and actors)’ [26].

Category 1 focuses on the macro-level of healthcare states and category 2 on the meso-level of organizations more generally. The categories 3 to 5 combine actor-centred and organizational dimensions, asking questions, like ‘who is responsible’ and at ‘what level of the organization’ [26]. Assessment of the meso-level organizational structure of hospitals is based on document analysis, expert information and published secondary sources.

This framework is expanded towards actor-centred perceptions. The selection criteria for the pilot research was to include two different perspectives on the dynamics of clinical management. The leading questions were: How do doctors working in patient care (the ‘managed’, case 1) perceive the organizational connections between medicine and management? How do doctors in management (the ‘managing’, case 2) perceive the organizational connections?

Case 1 draws on expert information and six in-depth qualitative interviews (semi-structured) with doctors working in four different departments in one large urban hospital carried out in 2013–15. The doctors selected for the interviews were specialists without a management position; the interviews focused on their perception of everyday work and management issues [39]. Case 2 uses material from semi-structured qualitative interviews with six department managers with a medical background working in hospitals in three different Swedish Counties. These interviews were selected for re-analysis from a larger study of 38 managers in different healthcare organizations in Sweden in 2007–08 exploring how healthcare managers (on different organizational levels and with different professional backgrounds) perceive leadership and management of doctors in their organizations [40, 41].

Data were analysed using a qualitative approach and thematic analyses with a focus on how the two groups of doctors describe the connections of medicine and management in their own work and organizational contexts. Including different Counties, organizational settings and points of time may help to disentangle structural gaps and weaknesses in clinical management from temporary artefacts of specific organizational failure.

## Results

### Hospital governance in Sweden: institutional dimensions of decision-making

In our assessment, we primarily focus on the meso-level of organizations and the operational dimensions of governance, following the five categories described previously.

1. *Key characteristics of the healthcare state and institutional contexts of hospital governance (macro-level)* are characterized by decentralized, partnership governance (strong inclusion of professional actors in regulatory bodies and policy-making) with little market but strong public control and patient involvement. Marketization is linked to patient choice thus reflecting a culture of equity and quality. Healthcare is tax-funded and the responsibility of the County Councils, the regional governments. The Councils have high control over funding and defining the operational dimensions of governance; they are also the owners of (most) hospitals.
  Hospital governance is characterized by complex forms of integrated governance with high levels of negotiations and more plural stakeholders. The trade unions of the medical profession are traditionally an important and powerful collective actor to negotiate framework agreements for salaries, while flexibility and relevance of individual negotiations are increasing. The vast majority of doctors, like other health professionals, are employees in hospitals [32, 33, 35, 42–44]. Like in other healthcare systems, mixed forms of hospital governance are increasingly gaining ground [1, 2].
2. *Governance structures of the hospital (meso-level/organization)* show high variation of the implementation of legislative regulation and guidelines on management. This is a result of a strongly decentralized governance structure. There are, nevertheless, some major characteristics, which make the model of Swedish hospital governance unique and different from the types found in other European healthcare systems, including in the Nordic countries. This may also be a reason why Sweden is often missing from European comparative studies (e.g., [2, 5]).
  To begin with, (almost all) hospitals are publicly owned, financed and controlled through a board appointed by the responsible County Council. On the top-level of the organization we usually find one executive manager who may be a doctor or another health professional. Despite some similarities with a clinical directorate, as found in the NHS systems for instance, there are important differences, including the attempt to rigorously open high-level leadership and management positions to all clinicians and to disentangle management responsibilities from (medical) professional power, at least on a legislative and formal level. This makes management more integrated and based on competences and organizational needs than on professional education. Related to this, there is also no position of a general manager.
  At the department level, the tasks of mangers are law-regulated with responsibility for budgets and, if they have the competence, for clinical management. In case the department manager is not a doctor, decision-making on medical issues will be delegated to a doctor. Furthermore important is the integration of the management responsibilities for doctors and nurses at this level, which makes the model different from the more pillarized structure of the troika types found in many other European countries [2, 26, 42].
  Overall, nurses usually have a strong position in middle- to lower-levels of management, which may to some extend include management responsibilities for doctors. However, on the first-line managerial levels (lower-level management) the professional background is gaining currency. Responsibility for nurses and doctors is becoming more separated, as a pillarized structure of medicine and nursing in lower managerial levels is emerging since about a decade or so. A major structural change has been the introduction of a new position of an ‘operational manager’, which is a doctor with management responsibilities below the department level.
  On the one hand, the new role of an operational manager may adapt the management structure of doctors to those of the nurses. On the other hand, there are important structural differences. The nurses are ward- or unit-based and therefore fit an organizational management structure, while the position of doctors is to a lesser degree defined by organizational categories (e.g., a doctor may be responsible for more than one unit/ward). There is also much variety and an overall lack of clear tasks, responsibilities and organizational resources of the doctors in this new management position. While some of these managers have clinical and administrative responsibilities, others are only responsible for clinical issues, for instance. Variety occurs between and within hospitals.
  This new position has created a controversial debate within the medical profession [45] (see further comments on sjukhuslakaren.se). It also seems that strengthening the position of doctors and introducing a professions-based management structure does not sit easily with an existing model of more integrated management and needs further investigation.
3. *Financial/efficiency controls and managerial tools (organizational levels, accountability and actors)* are mainly set by County Councils and are therefore highly diverse. Overall, resource allocation models are becoming increasingly more mixed, mainly based on fixed prospective per-case payments (based on DRGs), and complemented with price or volume ceilings and quality components ([35], p24). Approximately 50 % of all admissions in acute somatic care are estimated to be reimbursed by DRGs [33, 44].
4. *Quality and safety controls and organizational-managerial tools (organizational levels; accountability and actors)* strongly intersect with professional self-regulatory competencies and tools. Major responsibilities on the side of management include bottom-up controls with integrated medical power on all levels, for examples monitoring systems, patient safety procedures, and quality reports linked to financial incentives.
5. *Professional/medical self-governing controls and tools (organizational levels; accountability and actors)* are important and strongly integrated on all levels. For instance, performance indicators are increasingly relevant (among others to reduce high variation between hospitals and Counties) and systematically developed in collaboration with the National Board of Health and Welfare.

In what follows we explore the connections between medicine and management from the perspective of the doctors working at different sides of management.

### Case I: Doctors meet the organization

This case study looks at the doctor as an employee trying to understand the embeddedness of medicine–management relationships in organizational contexts at the micro-level of the hospital. As described previously, a new level of management was established between the department and the unit/ward attempting to improve accessibility of the manager in the day-to-day work of doctors.

The attempt to improve management met with mixed feelings on the side of the doctors. While the operational manager is more easily accessible, there was uncertainty about the tasks and concern about decision-making power. The doctors in this study clearly revealed a need for organizational support but avoided calling for a manager. Instead, they repeatedly used examples of inappropriate workload and inefficient work and worktime arrangements to highlight major problems and constraints of their everyday work, where they wished for a more efficient management. Similarly, the doctors often expressed a feeling of being without support in their everyday work but did not explicitly address this as an organizational demand and did not specify the support they are wishing for: ‘That you feel that there is some support from somewhere before it becomes a disaster….’ (#C).

Despite the organizational attempts to facilitate the connections, these doctors did not perceive their manager as an efficient structural link between themselves and the organization. They felt that their managers had little influence over their employees work situation, as one of the interviewees explained: ‘My closest manager, she is a very fine person, indeed, but I think…she has no mandate, and she has no time to discuss policy issues and things like that’ (#D).

The problem of ‘having no mandate’ was explained in various ways and in relation to different constraints. Hence, two patterns are emerging. First, the lack of decision-making power was seldom linked to the organizational structure but to personal attitudes. This includes, among others, the demand for a manager who ‘has the courage to stand up for the decisions he or she makes’ (# D) as well as disappointment that ‘many managers do not go against higher academic qualifications’ (# F). Second, and most interestingly, decision-making was rarely described in terms of hierarchical constraints but in relation to other professional groups, especially nurses.

[My manager] no, she hasn’t got more influence than anybody else here. The ones with the influence are the nurses. And the nurses at the ward. Because they are the ones, who interrupt the daily work the most. My manager doesn’t do that. (#D)

We express our opinions to our manager, and she agrees, and she brings our opinions to the manager for the nurses. And then she comes back and says: ‘No, it didn’t work’. (#C)

In summary, two interesting dimensions emerge from ‘the meeting of doctors and the organization’, namely the need for a manager taking care of doctor-employees and the demand for a manager who can make decisions, including in relation to nurses’ interests and responsibilities. The first dimension brings a demand for a medical manager into sight, who is different from the type of ‘nice and hard-working colleague’. This manager should be capable of doing the ‘core business’ of human resources management (HR), such as workplace issues, employer–employees relationships, and interprofessional coordination and decision-making. The second dimension reveals demand for organizational structures and routines, which clearly define responsibilities and roles and clarify accountability issues.

On this backdrop, the introduction of a new manager for doctors may not effectively respond to the needs of doctors and even create new problems, because of a coordination gap between the management of nurses and those of doctors may emerge, especially in the wards/units. Consequently, the situation reveals an organizational failure to develop efficient management models with clear definitions of tasks and decision-making procedures.

### Case II: Medical managers meet the organization

The second case focuses hospital doctors in the role of a manager. As described elsewhere, the manager role in healthcare organizations, regardless of the managers’ basic profession, was perceived as weak and even absent, and overall ambiguous in relation to the medical profession [40, 41]. When selecting the statements of the hospital managers with a medical professional background, we found an even stronger picture of an ambiguous manager role. Moreover, it seems that the perception of poor organizational support is stronger in this group compared the entire sample [40]. This perception is relevant in relation to the doctors the medical managers are supposed to lead, in relation to the organizational context, and in relation to the managerial system in which the job is performed.

The medical managers apparently are stuck in a ‘lonely wolf’ situation in the organization, which seems to be more generally an effect of hybridization of medicine and management. In search of support and advice on how to perform the manager role and the new tasks, they are foregrounding medical professional skills, while the (existing or potential) support tools and structures of the organization are not considered. The following quote clearly illustrates the complexity of the new ‘hybrid’ medical manager role, both in relation to the organizational context and in relation to the doctors who this manager is supposed to lead.

Managing physicians is extremely difficult … they really don’t want to be managed. … I’m actually a specialist in [other medical specialty not present at this clinic], I mean that I couldn’t go out there in the ward and say something like “All right my friends, you have to stop doing that”. That would probably not be accepted very well at all. And even if I did have the same specialty as they do … if I can’t point to a systematic review or something like that, showing that this, this isn’t good… You know, it’s part of the physician’s role to decide and choose what you yourself think is right …as department manager it’s pretty hard to influence this, actually very difficult. You have to stand on an immensely solid base to do that (#A). [41]

This example illustrates that a medical manager translates elements of professionalism and the medical culture of problem solving into management tasks. Thus, a figure of a ‘lonely wolf’ is created, who is personally very strong and who uses scientific evidence to solve problems. At the same time, this example points towards important gaps in the manager position that lacks acceptance within medicine. Consequently, from a clinical manager’s perspective, he is not capable of doing the job of management effectively.

And I think that some colleagues feel that they do not know how to do, they have no support, they do not have my support, they don’t have the patients’ support, they get no help from collaboration partners, so they’re very lonely. (#A)

Although the manager is aware of the dilemma, he does not see it as an organizational risk. He also does not ask for advice from the organization but seeks to solve the situation individually by being ‘immensely solid’.

Interestingly, this manager refers to medical specialties to explain the lack of acceptance of management in the medical profession, because doctors defend their own terrain against other ‘business’. While this pattern is relevant, there are fundamental differences in relation to the struggles between medical specialists. This manager does not enter the field as specialist in management, who would defend their competencies against other specialties. Instead, he accepts not to be competent in this area, and this in turn, furthers the devaluation of major tasks of his job in order to maintain an identity of a successful professional and an ‘immensely solid’ person.

Another pattern of devaluation of managerial competencies and roles appears in the figure of an ‘administrator’, as demonstrated by a manager cited below. In this case, medical knowledge served to outflank the organizational hierarchy, if the higher-level manager is not a doctor.

[He is] an administrator… who sorts the paper and looks at the money. …above me, there is no medical knowledge whatsoever. And also, really no knowledge of everyday life. They see the numbers and they get letters from the authorities, but the medical knowledge is at medical levels below. (#D)

When asked for the administrative responsibility of a clinical director, he explained: ‘Yes, I have, of course. …I unite the two parts, but above me, it’s just one part. There it is money and possibly politics’ (#D).

This construction of the doctor in management as positioned against the ‘administrator’ brings a persisting gap between medicine and management and a lack of accountability to the organization into sight, which may block efficient hospital management. The doctors also showed a desire to foreground their medical/clinical identities, thereby contributing to making the manager role invisible in the organization [41].

## Discussion

Clinical management in Sweden represents a type of complex ‘integrated governance’ with coordination between the levels and the substance (financial and quality/safety issues) of management, according to the typology developed in a European comparative assessment of hospital governance [26]. It is helpful to explore the organizational change in a wider context of hospital governance and to recall the importance of coordination as a taxonomy for exploring changes in clinical management [26].

The establishment of a new managerial position of a medical manager in lower management promotes separation of the management of doctors and nurses in a system, that otherwise shows structural conditions of integration and coordination and limited relevance of professional backgrounds in clinical management. This development mirrors a trend towards mixed governance as observed in European public hospitals [2], because elements from a ‘troika’ system with a pillarized management structure based on the professional groups are mixed with a more integrated model. However, it remains to be seen whether this is a temporary pattern or will become an element of Swedish hospital governance.

These conditions make the Swedish example an interesting case for an in-depth analysis, next to its experience with clinical management over time. It allows us to explore the ‘meeting’ of doctors and the organization in a situation where some action has been taken top-down on the organizational level to respond to changing management needs of the rank-and-file doctors through structural changes.

The results show that structural change does not easily translate into micro-level changes. Medical managers are primarily perceived as colleagues and therefore not asked for advice on managerial issues. This perception is problematic for various reasons: it leaves the doctors working in patient care without appropriate management support. This situation may reinforce pressure, disappointment and dissatisfaction; and it may turn out as devaluation of management competencies of medical managers in relation to their professional expertise, as some of the interviewees have illustrated.

On the other hand, the managers themselves do not adequately rely on their management competencies but on personal strength, thus creating a figure of a ‘lonely wolf’ lacking organizational support. The medical managers do not primarily define themselves as managers and part of an organizational system, and they do not refer to management tools to solve problems. This perception points to an organizational failure to create supportive institutional environments and structural adjustment to new management roles, for instance by offering specialty training for managers, supervision, advisory boards for high-profile medical managers, or so. Even if most hospitals today offer management training programmes, these programmes obviously do not provide sufficient support for medical managers.

Bringing doctors into management, therefore, may create hybrid *concepts* of management but not necessarily supportive organizational environments for medical managers to exercise their new role more efficiently. The mere introduction of new roles does not guarantee closer connections and better management.

Another important finding is the relevance of professionalism and medical identities, which do not ‘hybridize’ as easily as discourses may suggest. It is therefore important to connect organizational and professional innovation more systematically [3, 23]. The medical profession has not kept pace with the management need of their members, whether they are working with patients or as doctors in management. Doctors seem to use medical knowledge as a means of problem solving and a yardstick for competence. As managers they translate this strategy to new management tasks, and for doctors in care the medical competence of their managers overrides any managerial weakness and lack of power of doctors in management in the organization.

These results might point towards important gaps in medical education that cannot be sufficiently solved on the organizational level of governance and need further attention to better connect health and educational systems [38, 46]. Furthermore, the individual coping strategies of doctors show that the power of medical knowledge is highly flexible and capable of serving changing demands without necessarily weaken its power [11, 47].

### Methodological limitations

The explorative nature of the study and the use of small cases as pilots to bring micro-level effects into view has several limitations and clearly calls for caution with drawing general conclusions. There is some plausibility that identifying management gaps and organizational weaknesses in an integrated healthcare system like Sweden points towards more general problems with connecting medicine and management, but the findings must be tested in empirical settings in other healthcare systems. Furthermore, the evidence of the needs and demands of doctors is still weak and lacks connection with organizational indicators and efficiency measures. Further research and survey data are therefore necessary to develop organization interventions and monitor the effects.

## Conclusion

This study has aimed to look inside the box of hospital governance. It adds new knowledge to a growing body of research into medical management – mainly concerned with financial effects and policy and sometimes with service approaches [48] – by applying a multi-level governance approach and bringing the needs of doctors into view. The results show that integrated modes of hospital governance on the marco- and meso-levels, like in the Swedish system, do not easily impact further down on the micro-level in ways that create efficient organizational responses to the needs of doctors. Doctors in managerial positions do not automatically create qualitative improvements in the management–medicine coordination, while hospital organizations do not effectively support their medical managers. This study brings gaps in hospital governance into view that may create organizational weaknesses and unmet management needs, thereby constraining more coordinated and integrated medical management.

### Ethical approval

Material used in Case 1 (managed doctors) did not need ethical approval according to Swedish law; material used in case study 2 (medical managers) was part of a larger research, which was approved by the Regional Ethics Committee of Stockholm (2006/1293-31).
